# Factors associated with occurrence of salmonellosis among children living in Mukuru slum, an urban informal settlement in Kenya

**DOI:** 10.1186/s12879-020-05134-z

**Published:** 2020-06-17

**Authors:** Cecilia Mbae, Moses Mwangi, Naomi Gitau, Tabitha Irungu, Fidelis Muendo, Zilla Wakio, Ruth Wambui, Susan Kavai, Robert Onsare, Celestine Wairimu, Ronald Ngetich, Frida Njeru, Sandra Van Puyvelde, John Clemens, Gordon Dougan, Samuel Kariuki

**Affiliations:** 1 Centre for Microbiology Research, Kenya Medical Research Institute, Off Mbagathi Road, PO Box 54840-00200, Nairobi, Kenya; 2 Centre for Public Health Research, Kenya Medical Research Institute, Nairobi, Kenya; 3 Department of Medicine, University of Cambridge, Cambridge, UK; 4 Department of Biomedical Sciences, Institute of Tropical Medicine, Antwerp, Belgium; 5 Laboratory of Medical Microbiology, Vaccine & Infectious Disease Institute, Universiteit Antwerpen, Antwerp, Belgium; 6 International Centre for Diarrhoeal Disease Research, Dhaka, Bangladesh; 7 Wellcome Sanger Institute, Wellcome Genome Campus, Hinxton, Cambridge, UK

**Keywords:** Invasive salmonellosis; children, Socioecomic, Environmental, Risk factors, Informal settlement, Nairobi, Kenya

## Abstract

**Background:**

In Kenya, typhoid fever and invasive non-typhoidal salmonellosis present a huge burden of disease, especially in poor-resource settings where clean water supply and sanitation conditions are inadequate. The epidemiology of both diseases is poorly understood in terms of severity and risk factors. The aim of the study was to determine the disease burden and spatial distribution of salmonellosis, as well as socioeconomic and environmental risk factors for these infections, in a large informal settlement near the city of Nairobi, from 2013 to 2017.

**Methods:**

Initially, a house-to-house baseline census of 150,000 population in Mukuru informal settlement was carried out and relevant socioeconomic, demographic, and healthcare utilization information was collected using structured questionnaires. *Salmonella* bacteria were cultured from the blood and faeces of children < 16 years of age who reported at three outpatient facilities with fever alone or fever and diarrhea. Tests of association between specific *Salmonella* serotypes and risk factors were conducted using Pearson Chi-Square (χ^2^) test.

**Results:**

A total of 16,236 children were recruited into the study. The prevalence of bloodstream infections by Non-Typhoidal *Salmonella* (NTS), consisting of *Salmonella* Typhimurium/ Enteriditis, was 1.3%; *Salmonella* Typhi was 1.4%, and this was highest among children < 16 years of age. Occurrence of *Salmonella* Typhimurium/ Enteriditis was not significantly associated with rearing any domestic animals. Rearing chicken was significantly associated with high prevalence of *S.* Typhi (2.1%; *p* = 0.011). The proportion of children infected with *Salmonella* Typhimurium/ Enteriditis was significantly higher in households that used water pots as water storage containers compared to using water directly from the tap (0.6%). Use of pit latrines and open defecation were significant risk factors for *S.* Typhi infection (1.6%; *p* = 0.048). The proportion of *Salmonella* Typhimurium/ Enteriditis among children eating street food 4 or more times per week was higher compared to 1 to 2 times/week on average (1.1%; *p* = 0.032).

**Conclusion:**

Typhoidal and NTS are important causes of illness in children in Mukuru informal settlement, especially among children less than 16 years of age. Improving Water, Sanitation and Hygiene (WASH) including boiling water, breastfeeding, hand washing practices, and avoiding animal contact in domestic settings could contribute to reducing the risk of transmission of *Salmonella* disease from contaminated environments.

## Background

In sub-Saharan Africa (SSA) non-typhoidal *Salmonella* (NTS) is a major cause of invasive bacterial infections in infants and young children, the elderly, immunocompromised and the malnourished [1–6]. Invasive NTS disease is caused mainly by *Salmonella enterica* subspecies *enterica* serovars Typhimurium and Enteritidis [6–9]. Multi-drug resistant (MDR) iNTS is common in resource-poor settings in Kenya [10], and in other parts of SSA [11–16] posing a major challenge to treatment and management options available. In studies on NTS in Kenya Tabu et al., (2012) documented crude incidence of 568/100,000 person-years of observation (pyo) in a rural site near Kisumu (Western Kenya) and 51/100,000 pyo in an urban informal settlement, Kibera, which is one of the major informal settlements in Nairobi. However, true rates in both sites were thought to be underestimated by 4–8 fold due to insensitivity of blood cultures (55–60%) to detect bacteremic infections.

Typhoid fever, caused by *S*. Typhi is also endemic in SSA, partly because the supply of clean drinking water and sanitation have not kept pace with the rapid population growth [17]. Typhoid is now estimated to have an average annual incidence of 263/100,000 pyo (95% CI: 199–347) in all age groups in Kenya [18] and causes more illness among older children compared to NTS [6]. These rates are very similar to what has been documented in slums in Pakistan [19], India [20, 21] and Bangladesh [22]. The MDR-associated *S*. Typhi H58 clade is now widely disseminated in East Africa [8, 23–26]. In Kenya, MDR *S*. Typhi H58 has been associated with sporadic outbreaks involving adults and school-age children living within in resource-poor settings [25]. Unlike iNTS, HIV is not regarded as a major risk factor for typhoid fever [10].

In the informal settlements around Nairobi, epidemiology of typhoid and iNTS disease, transmission dynamics, risk factors and, the circulating serotypes and genotypes are not well understood. It is therefore important to carry out detailed surveillance to obtain data on temporal changes of *Salmonella* serotype diversity, risk factors and variations in their antimicrobial susceptibility profiles, which are crucial for informing clinical care, updating treatment guidelines, and guiding public health interventions.

More than 34% of Kenyans live in urban areas, with more than 50% living in Informal Settlements that constitute only 5% of the residential area [27]. The informal settlements have limited access to clean water, sanitation facilities, solid-waste management, drainage, and electricity [28]. These factors likely contribute to a high incidence of diarrheal diseases and mortality among children [29, 30].

A major challenge in addressing the issues of managing both NTS and typhoid in our settings is emerging resistance to most commonly used antimicrobials, and now to even reserve antibiotics [6, 8]. For instance, ESBL producing *S*. Typhimurium and *S*. Typhi with reduced susceptibility to fluoroquinolones pose a huge challenge in management of severe salmonellosis in Kenya.

The aim of this study was to determine the incidence, spatial distribution, socioeconomic and environmental risk factors for Salmonella infections in Mukuru informal settlement, one of the largest sprawling informal settlement 20 km east of Nairobi. In this paper, we report on a 5-year study of population-based surveillance for iNTS disease, typhoid fever, and NTS diarrhea, in children under 16 years of age.

## Methods

### Mapping Mukuru informal settlement site

Mukuru informal settlement is located 20 km east of Nairobi city, with a population of around 700,000 people [31] and is divided into eight villages; Mukuru Lunga-Lunga, Mukuru Sinai, Mukuru kwa Reuben, Mukuru kwa Njenga, Mukuru Kayaba, Kosovo and Mukuru North. Our study was carried out in two of the villages, Mukuru kwa Njenga and Mukuru kwa Ruben, which have a population of approximately 150,000. In the study area, families live in corrugated iron huts measuring ca.10 ft. x10ft, and large families (4–8 members) are crammed into this tiny space. During the rainy season storm drainage and sewer water runs around these shelters. The overcrowding and the lack of proper sanitary facilities likely contribute to the rapid spread of enteric infections. The residents obtain water from common watering points supplied by the City Council, each serving close to 1500 residents. In addition, several vendors hawk this water to residents.

A house-to-house baseline census was carried out and relevant socioeconomic, demographic, and healthcare utilization data was collected. Name and age of each member in the family and other members in the household was recorded so as to give the target list in the area for subjects. The census was preceded by an intensive campaign to publicize the study.

High resolution satellite imagery technology was used to create Household-level geographic information system (GIS) database. An image was acquired from Google Earth to create the map of structures/buildings of the study area as the first step of creating the GIS database. The satellite images were enhanced using image processing software before houses/buildings were digitized. GPS was used to capture data at several identifiable points on the images that would be used as ground control points (GCPs). The images were geometrically rectified to a known coordinate system, Universal Transverse Mercator (UTM) 37S, on the basis of a number of GCPs. After geo-referencing the images were resampled, and converted into TIFF files in ArcGIS10x software [32].

Considering the dynamic nature of the population sizes in slum areas, we fused use of high resolution satellite images to delineate houses, used local boundaries to associate the houses by roof, and ground truthing by way of household survey to geo-locate the population [33, 34].

### Block identification, numbering and hard copy labeling

Mukuru kwa Ruben was divided into nine zones while Mukuru Kwa Njenga was divided into eight zones for purposes of Digitization and grouping blocks into individual zones (Table 1). The base map data used was high resolution Orthophoto aerial photographs in WGS84 Zone 37S Projected UTM Coordinates and analysed using Arc Map version 10.1. Total ground area of the study was 324.22 ha. The zone with the biggest area was Riara Zone with 51.67 ha while the smallest area was Bins Zone with 4.08 ha.

**Table 1:** Mukuru villages Zones, area and number of Blocks per Zone

Name	Zone No	Area (Ha)	No. of Blocks Labeled
**Mukuru Kwa Ruben Zones**			
Bins Zone	1	4.08	406
Mombasa Zone	2	5.97	691
Feed the Children Zone	3	5.84	507
Gatoto Zone	4	6.95	551
Rurii Zone	5	23.21	1003
Kosovo Zone	6	13.09	775
Falcon Zone	7	13.86	1026
Railway Zone	8	21.85	1425
Simba Cool Zone	9	18.62	675
**Mukuru Kwa Njenga Zones**			
Riara Zone	1a	51.67	–
MCC Zone	2a	27.8	–
Motomoto Zone	3a	14.28	876
Sisal Zone	4a	38.13	2881
Vietynam Zone	5a	30.43	1409
48 Zone	6a	25	1234
Milimani Zone	7a	9.28	814
Wape Wape Zone	8a	14.16	847

**Fig. 1:**
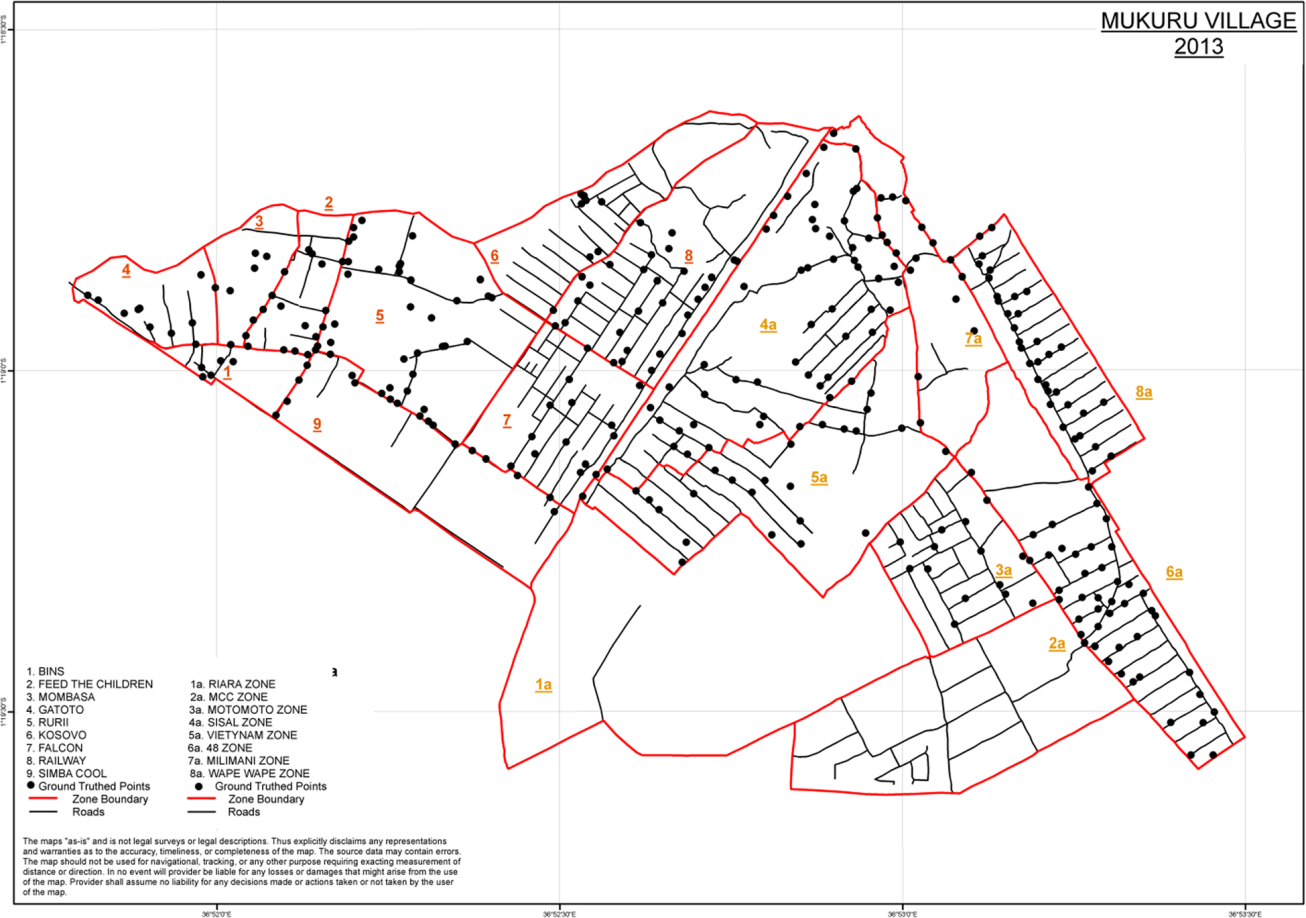
Two study areas in Mukuru (Mukuru Kwa Ruben and Mukuru Kwa Njenga) showing mapped zones. The map shows study area zone boundaries in Mukuru Njenga and Mukuru Reuben, Nairobi, Kenya. The boundaries and road tracks were mutually agreed with local authority and later digitized against a backdrop of June 2013 Google Earth Imagery on ArcGis 10.2. The ground –truthed points were collected by hand held GPS to depict locations of social amenities important in the study

All the households, zones, roads and the outlining boundary of the region were digitized with each forming a shape file for the entire study area (Fig. 1). A high resolution hard copy A0 map with background orthophotos and zone boundary was printed for both Mukuru Kwa Ruben and Njenga regions. This facilitated fast labeling of the blocks where block numbers were labeled per zone to allow for the household census to start (Table 1). Zone 3a and 4a were the only ones labeled whereas zones 1a and 2a were excluded from the study area as they were of urban residential settlements. The household units as viewed from the printed aerial photos were numbered so as to facilitate the survey process which was to be carried out. The numbers on the map were later synchronized with the field Identity Numbers corresponding to the digitized households/plots so as to match the map, survey and the database.

### Surveillance for salmonella disease

Patients were recruited from the three health care facilities serving the population, including Medical Missionaries of Mary, Reuben Centre and City council clinic in Embakasi. Inclusion criteria for cases included; children < 16 years of age on the date of presentation who reside within Mukuru informal settlements, they presented with a subjective history of at least 3 days of fever and with axillary temperature of at least 38 °C or had history of fever of any duration with an axillary temperature of at least 39 °C and/or reported three or more loose or liquid stools in the 24 h before presentation, or one or more loose or liquid stool with visible blood. Eligible patients whose guardians provided a written informed consent had detailed history and physical examination recorded on a structured data form. Febrile patients had blood and stool (either whole stool or rectal swab) samples taken for culture and antibiotic susceptibility testing. All patients had blood taken for evaluation for malaria, HIV, and presence of sickle cell trait/disease. A transmittal form was used to track movement of samples from clinic to laboratory. A study log was kept at each clinic site, noting all potentially eligible patients, their presenting symptoms, whether or not they participated, reasons for non-participation, and whether blood specimens for culture, malaria smear, haemoglobin electrophoresis, and HIV testing and a faecal specimen for culture were collected and tested.

A total of 16,236 patients were recruited into the study. From eligible patients, stool specimen was collected in sterile sampling cups, part of the specimen was aliquoted using sterile cotton swabs in Cary Blair Transport Medium (Oxoid Ltd., Basingstoke, UK). The samples were then transferred to the laboratory at the Centre for Microbiology Research (KEMRI) within 4 h. A structured questionnaire was used to elucidate the following information from each diarrhea case and healthy control after the stool samples were collected: clinical manifestations (e.g. vomiting, fever, and/or dehydration), demographic data (age, sex, and residence), and types of stool samples (watery, mucous, or bloody, or other form). For blood culture 1–3 ml for children < 5 years of age and 5–10 ml for 5–16 years of age was collected in syringe, placed into Tryptic Soy Broth (TSB) media in Bactec bottles, and transported daily to and analyzed at the KEMRI laboratory.

### Laboratory analysis

#### Blood for culture

Blood cultures were incubated at 37 °C in a computerized BACTEC™ 9050 Blood Culture System (BD, Franklin Lakes, New Jersey, USA), and subcultured after 24 h onto blood, chocolate and MacConkey agar plates. The blood cultures were subsequently observed for a further 7 days for signs of bacterial growth (auto-detection). A final subculture was performed for all blood cultures on the 8th day regardless of the state of bacterial growth. From the subcultures, bacterial isolates were identified using biochemical tests on API20E strips (API System, Montalieu Vercieu, France) and further typed by species-specific serological tests.

#### Stool cultures

The rectal swab or loopful of the stool specimen was transported to KEMRI laboratory and initially cultured on selenite F (Oxoid, Basingstoke, UK) broth aerobically at 37 °C overnight. Broth cultures were then subcultured on MacConkey agar and *Salmonella-Shigella* agar (Oxoid) and incubated at 37^o^ C overnight. To identify suspect *Salmonella* bacteria, non-lactose fermenting colonies were biochemically tested using triple sugar iron (TSI) slants. From the subcultures, bacterial isolates were identified using biochemical tests on API20E strips and further typed by species-specific serological tests (Remel, Thermo Fisher Scientific, MA, USA).

##### Antimicrobial susceptibility testing

Antimicrobial susceptibility testing was performed using the disk diffusion technique for all commonly used antimicrobials in Kenya on Mueller-Hinton agar (Oxoid, Basingstoke, UK). Antimicrobial agents tested included; ampicillin 10 μg, tetracycline 30 μg, gentamicin 10 μg, trimethoprim 5 μg, sulphamethoxazole 100 μg, chloramphenicol 30 μg, co-amoxiclav 20:10 μg, cefuroxime 30 μg, ceftazidime 30 μg, ceftriaxone 30 μg, cefotaxime 30 μg, ciprofloxacin 5 μg and nalidixic acid 10 μg. Determination of the minimum inhibitory concentrations of the antimicrobials was performed using the E-test strips (AB BIODISK, Solna, Sweden). Results were interpreted according to the guidelines provided by the Clinical and Laboratory Standards Institute (CLSI) (2017).

#### Patient information and care

After laboratory analysis of the samples, results were taken back to the attending clinician for patient management. Those that were found to be positive for salmonella infection were contacted on telephone, and asked to report to the clinic, where the results were communicated to them. The clinician then provided a drug prescription based on the antimicrobial susceptibility test results. The patient/guardian was given details on the importance of taking and completing the drug dose, and health information on the disease, and how to prevent future infections. All patients were given water treatment tablets after the health education. The patient/guardian was then handed over to a community health worker (CHW), who would follow the patient to their homes and fill in a questionnaire on socio-demographic characteristics of the household and assess other environmental risk factors for enteric diseases. The questionnaire collected information including; demographic and socioeconomic features of the households: age and sex of all residents, household size, income, ownership of luxury items, and education, the source of water used for drinking and washing, hand washing location, presence of hand soap at the hand washing location, use of refuse containers, presence of visible stool in the yard or home, types of water storage containers used in the home, site(s) and structure(s) used for defecation, household food preparation methods, out of household food exposures, antibiotic use during the 2 weeks prior to presentation of the case, and presence and number of different domesticated animals in the house. The CHW also followed up the patients to ensure that they have reported back to the clinic for stool culture re-testing after completion of the treatment.

#### Data analysis for risk factors

Statistical analyses were performed using SPSS software version 25.0. Descriptive statistics were presented as counts and percentages. Tests of association between specific salmonellosis and risk factors were conducted using Pearson Chi-Square (χ^2^) test. Odds ratio and their corresponding confidence interval (95% CI) were used to measure the strength of association. The threshold for significance was set at 0.05 for all tests. All significant risk factors (*p*-value < 0.05) were adjusted for confounders and risk modifiers using multivariable binary logistic regression and specifying backward conditional as the removal method. Reduced model showing adjusted odds ratio, their corresponding confidence intervals (95%CI) and *p*-values were reported.

#### Ethics approval and consent to participate

Written informed consent to participate was obtained from the parents/guardians of the minors included in this study (minors were defined as anyone under the age of 16 years). Ethical approval was granted by the Scientific Ethics and Review Unit (SERU) of the Kenya Medical Research Institute (KEMRI) (SSC. No. 2076), and from the County Health Executive and Ministry of Health, Embakasi subcounty.

## Results

### Selected demographic characteristics and salmonella disease

A total of 16,236 children were recruited into the study. The prevalence of *Salmonella* Typhimurium/ Enteriditis from blood and stool of patients with fever was 1.3% (CI: 1.1–1.5%), while that of *S.* Typhi was 1.4% (CI: 1.2–1.6%). There was a comparable male (51.1%) to female (48.9%) while most of the children (63.0%) were aged less than 5 years, constituted by 39.5% aged 0 to 2 years and 23.5% aged 3 to 4 years. Table 2 presents the distribution of children in the study by selected demographic characteristics.

**Table 2:** Distribution of selected demographic characteristics

Variables	*N* = 16,236	%
**Gender of the child**		
Male	8296	51.1
Female	7940	48.9
**Age of the child**		
0–2 years	6408	39.5
3–4 years	3807	23.5
5–6 years	1980	12.2
7–8 years	1339	8.3
over 8 years	2694	16.6
Missing	8	

The occurrence of *Salmonella* Typhimurium/*E*nteriditis was not significantly associated with the selected demographic characteristics presented (Table 3). However, males were significantly associated with high prevalence of *S.* Typhi (1.8%; OR = 1.49 (CI:1.15–1.95); *p* = 0.003) compared to females (1.2%). A high proportion of infection with *S.* Typhi was observed among children aged 5–6 years (2.6%; OR = 2.45 (CI:1.68–3.56); *p* < 0.001) and 7–8 years (2.1%; OR = 2.00 (CI:1.27–3.15); *p* = 0.003), compared to children aged 0–2 years (1.1%).

**Table 3:** Salmonella disease in relation to selected demographic characteristics

	*Salmonella* Typhimurium/Enteriditis				*Salmonella* Typhi		
	N	Pos (%)	OR^a^(95%CI^b^)	*p* value	Pos (%)	OR^a^(95%CI^b^)	*p* value
**Gender of the child**							
Male	7861	1.40%	0.93(0.71–1.21)	0.57	1.80%	1.49(1.15–1.95)	**0.003**
Female	7487	1.50%	Ref		1.20%	Ref	
**Age of the child**							
0–2 years	5992	1.30%	Ref		1.10%	Ref	
3–4 years	3607	1.60%	1.21(0.85–1.70)	0.277	1.50%	1.41(0.98–2.03)	0.066
5–6 years	1903	1.40%	1.06(0.69–1.65)	0.783	2.60%	2.45(1.68–3.56)	**< 0.001**
7–8 years	1277	1.60%	1.18(0.72–1.93)	0.52	2.10%	2.00(1.27–3.15)	**0.003**
over 8 years	2561	1.30%	0.99(0.66–1.49)	0.978	1.50%	1.43(0.96–2.14)	0.079

^a^*OR* Odds Ratio. ^*b*^*CI* Confidence interval

### The influence of domestic animals on salmonella disease

The most common animals included cats (23.2%), chicken (18.0%) and dogs (11.5%), whereas other animals accounted for less than 10%. Table 4 presents the distribution of homesteads by specific domestic animal kept, water, hygiene, sanitation and source of food. The majority of the households in the settlement use a communal tap (78.5%), with 75.2% reporting the existence of contamination sources within 20 m of the water source. The most commonly used water storage containers in the home included Jerri cans (38.6%) and water pots (37.6%), with 40.6% of the households never boiled water before drinking. Most of the households (67.4%) used public toilets while 28.0% used flushing toilets. The majority (81.7%) of the households reported that they washed their hands after defecation.

**Table 4:** Distribution of homesteads by specific domestic animal kept, water, hygiene, sanitation and source of food

Variables	*N* = 16,236	%
Sheep	451	2.8
Cattle	639	3.9
Dogs	1868	11.5
Pigs	980	6
Cats	3774	23.2
Chicken	2921	18
Goats	1018	6.3
Other	335	2.1
**Main source of drinking water in the house**		
Own tap	1113	6.9
Own well	89	0.5
Communal tap	12,730	78.5
Communal well/pump	1938	12
River/Spring/Rainwater	52	0.3
Other	285	1.8
Missing	29	
**Existing contamination sources around the water source within 20 m** e.g. open sewers, communal latrines/toilets	12,129	75.2
**Types of water storage containers used in the home**		
Directly from tap	744	4.6
Water pot	6054	37.6
Pitcher	1809	11.2
Jerri can	6224	38.6
Other	1288	8
Missing	117	
**Water generally boiled before drinking**		
Always	6025	40
Sometimes	2929	19.4
Never	6069	40.3
Don’t know	46	0.3
Missing	1109	
**Type of toilet used by the Household**		
Public toilet	10,891	67.4
Flush toilet	4529	28
Pit latrine	661	4.1
Bush/river/canal	72	0.4
Don’t know	15	0.1
Missing	10	
**Household members wash hands after defecation**		
Always	13,211	81.7
Sometimes	2722	16.8
Never	115	0.7
Don’t know	118	0.7
Missing	70	
**Frequency of family eating street food**		
Never/rarely	3787	23.6
1 to 2 times/week	6109	38
3 to 5 times/week	3922	24.4
4 or more/week	2242	14
Missing	176	
**Grow in backyard**	205	1.3
**Buy from shop**	8247	50.8
**Buy from neighbor**	1397	8.6
**Buy from our mobile vendor**	4519	27.8
**Buy from village market**	4104	25.3

The occurrence of *Salmonella* Typhimurium/*E*nteriditis was not significantly associated with rearing any domestic animal (Table 5). However, rearing chickens and goats was significantly associated with a higher prevalence of *S.* Typhi (2.1%; OR = 1.75 (CI:1.15–2.70); *p* = 0.011; and (2.5%; OR = 1.49 (CI:1.15–2.00); *p* = 0.011), respectively.

**Table 5:** Salmonella disease in relation to keeping domestic animals

Variables	*Salmonella* Typhimurium/ Enteriditis				*Salmonella* Typhi		
	N	Pos (%)	OR^a^(95%CI)	*p* value	Pos (%)	OR(95%eCI^b^)	*p* value
**Sheep**							
Not present	14,931	1.40%	0.99 (0.44–2.24)	0.983	1.50%	1.05 (0.47–2.38)	0.893
Present	417	1.40%	Ref		1.40%	Ref	
**Cattle**							
Not present	14,755	1.40%	1.22 (0.57–2.6)	0.606	1.50%	0.81 (0.44–1.49)	0.495
Present	593	1.20%	Ref		1.90%	Ref	
**Dogs**							
Not present	13,582	1.40%	0.96 (0.64–1.46)	0.864	1.50%	0.78 (0.54–1.14)	0.202
Present	1766	1.50%	Ref		1.90%	Ref	
**Pigs**							
Not present	14,442	1.40%	0.92 (0.53–1.58)	0.757	1.50%	0.75 (0.46–1.21)	0.237
Present	906	1.50%	Ref		2.00%	Ref	
**Cats**							
Not present	11,773	1.50%	1.28 (0.92–1.8)	0.148	1.50%	0.93 (0.69–1.27)	0.672
Present	3575	1.20%	Ref		1.60%	Ref	
**Chicken**							
Not present	12,578	1.40%	0.85 (0.61–1.18)	0.333	1.40%	Ref	
Present	2770	1.60%	Ref		2.10%	1.75 (1.15–2.70)	**0.011**
**Goats**							
Not present	14,391	1.40%	1.05 (0.60–1.85)	0.854	1.50%	Ref	
Present	957	1.40%	Ref		2.50%	1.49 (1.10–2.00)	**0.011**
**Other**							
Not present	15,038	1.40%	1.11 (0.41–3.00)	0.838	1.50%	1.74 (2.68–1.14)	0.74
Present	310	1.30%	Ref		1.30%	Ref	

^a^*OR* Odds Ratio. ^*b*^*CI* Confidence interval

### The effect of water, hygiene and sanitation on occurrence of salmonella disease

The proportion of children infected with *Salmonella* Typhimurium/ *E*nteriditis was significantly higher in households that used water pots (1.6%; OR = 2.75(CI:1.01–7.51); *p* = 0.0048) and Jerri cans (1.6%; OR = 2.81(CI:1.03–7.66); *p* = 0.044) to store water compared to using water directly from the tap (0.6%) (Table 6). A similar trend was observed among households that use pit latrines (2.5%; OR = 1.77(CI:1.01–3.08); *p* = 0.045) compared to households using flush toilets (1.4%). Similarly, the use of bush/river/canal (5.6%; OR = 4.93(CI:1.73–14.03); *p* = 0.003) and pit latrines (1.6%; OR = 1.37(CI:1.00–1.89); *p* = 0.048) as toilets was associated with a higher proportion of *S.* Typhi infection among children compared to use of flush toilet (1.2%). The proportion of children infected with *S.* Typhi was significantly higher amongst households whose members never wash hands after defecation (4.5%; OR = 3.18(CI:1.28–7.90); *p* = 0.013) compared to households where they always practice hand washing (1.5%).

**Table 6:** Salmonella disease in relation to various predictors

**Predictors of*Salmonella*Typhimurium/Enteriditis**		
**Variables**	**aOR**^**a**^**(95% CI**^**b**^**)**	***p*-value**
**Frequency of family eating street food**		
Never/rarely	0.84 (0.55–1.29)	0.435
1 to 2 times/week	0.65 (0.43–0.98)	**0.041**
3 to 5 times/week	0.95 (0.62–1.44)	0.8
4 to 6 more /week	Ref	
**Types of water storage containers used in the home**		
Directly from tap	Ref	
Water pot	2.69 (0.98–7.37)	0.054
Pitcher	1.18 (0.38–3.69)	0.772
Jerri can	2.63 (0.96–7.19)	0.059
Other	2.13 (0.71–6.41)	0.178
**Predictors of*Salmonella*Typhi**		
**Variables**	**aOR**^**a**^**(95% CI**^**b**^**)**	***p*value**
**Gender of the child**		
Male	1.53 (1.17–2.01)	**0.002**
Female	Ref	
**Age of the child**		
0–2 years	Ref	
3–4 years	1.43 (0.99–2.07)	0.059
5–6 years	2.49 (1.70–3.65)	**< 0.001**
7–8 years	2.08 (1.31–3.28)	**0.002**
Over 8 years	1.43 (0.95–2.15)	0.083
**Keeping chicken**		
Present	1.33 (0.95–2.15)	0.071
Not present	Ref	
**Type of toilet used by the household**		
Public toilet	1.35 (0.98–1.87)	0.064
Flush toilet	Ref	
Pit latrine	1.53 (0.81–2.90)	0.193
Bush/river/canal	5.11 (1.79–14.62)	**0.002**
Don’t know	UD(N/A)	1

^a^*OR* Odds Ratio. ^*b*^*CI* Confidence interval* N/A not applicable*

### Household sources of food

The proportion of *Salmonella* Typhimurium/Enteriditis was significantly higher among children raised in households whose frequency of eating street food was 4 to 6 or more times compared to 1 to 2 times/week (1.1%; OR = 0.64(CI:0.43–0.96); *p* = 0.032). The occurrence of *S.* Typhi was not significantly associated with any specific source of food within the informal settlement (Table 7). A total of 38.4% of the families from the study area ate street food three or more times/week. Approximately half of these (50.8%) bought food from the shop, with 27.8% buying from mobile vendors and 25.3% from a village market as shown in Table 7. A relatively small number of households grew vegetables in the backyard (1.3%), with 8.6% buying from neighbours.

**Table 7:** Salmonella disease in relation to source of food

Variables	*Salmonella* Typhimurium/Enteriditis				*Salmonella* Typhi		
	N	Pos (%)	OR^a^(95%CI^b^)	*p* value	Pos (%)	OR^a^(95%CI^b^)	*p* value
**Frequency of family eating street food**							
Never/rarely	3594	1.50%	0.83 (0.54–1.27)	0.394	1.50%	1.03 (0.66–1.62)	0.899
1 to 2 times/week	5788	1.10%	0.64 (0.43–0.96)	**0.032**	1.50%	1.07 (0.71–1.63)	0.736
3 to 5 times/week	3707	1.70%	0.95 (0.63–1.43)	0.788	1.60%	1.11 (0.71–1.73)	0.636
4 to 6 more /week	2093	1.80%	Ref		1.40%	Ref	
**Growing in backyard**							
No	15,153	1.40%	0.69 (0.25–1.87)	0.462	1.50%	0.48 (0.22–1.08)	0.08
Yes	195	2.10%	Ref		3.10%	Ref	
**Buying from shop**							
No	7468	1.50%	1.17 (0.90–1.53)	0.251	1.40%	0.91 (0.71–1.178)	0.478
Yes	7880	1.30%	Ref		1.60%	Ref	
**Buying from neighbour**							
No	14,021	1.40%	1.00 (0.62–1.60)	0.987	1.50%	0.82 (0.54–1.27)	0.367
Yes	1327	1.40%	Ref		1.80%	Ref	
**Buying from mobile vendor**							
No	11,109	1.40%	0.83 (0.62–1.10)	0.196	1.60%	1.29 (0.95–1.79)	0.094
Yes	4239	1.60%	Ref		1.30%	Ref	
**Buying from village market**							
No	11,552	1.50%	1.21 (0.87–1.67)	0.259	1.50%	0.99 (0.74–1.33)	0.955
Yes	3796	1.20%	Ref		1.50%	Ref	

^a^*OR* Odds Ratio. ^*b*^*CI* Confidence interval

### Predictors of salmonella disease among children living in Mukuru informal settlement

Factors identified by bivariate analysis showing significant association with specific *Salmonella* serotype include the frequency of family eating street food, types of water storage containers used, gender and age of the child, keeping chicken and type of toilet used by the household. Table 8 presents the predictors of Salmonellosis among children living in Mukuru informal settlement.

**Table 8:** Predictors of salmonella disease among children

**Predictors of*Salmonella*Typhimurium/Enteriditis**		
**Variables**	**aOR**^**a**^**(95% CI**^**b**^**)**	***p*value**
**Frequency of family eating street food**		
Never/rarely	0.84 (0.55–1.29)	0.435
1 to 2 times/week	0.65 (0.43–0.98)	**0.041**
3 to 5 times/week	0.95 (0.62–1.44)	0.800
4 to 6 more /week	Ref	
**Types of water storage containers used in the home**		
Directly from tap	Ref	
Water pot	2.69 (0.98–7.37)	0.054
Pitcher	1.18 (0.38–3.69)	0.772
Jerri can	2.63 (0.96–7.19)	0.059
Other	2.13 (0.71–6.41)	0.178
**Predictors of*Salmonella*Typhi**		
**Variables**	**aOR**^**a**^**(95% CI**^**b**^**)**	***p*value**
**Gender of the child**		
Male	1.53 (1.17–2.01)	**0.002**
Female	Ref	
**Age of the child**		
0–2 years	Ref	
3–4 years	1.43 (0.99–2.07)	0.059
5–6 years	2.49 (1.70–3.65)	**< 0.001**
7–8 years	2.08 (1.31–3.28)	**0.002**
Over 8 years	1.43 (0.95–2.15)	0.083
**Keeping chicken**		
Present	1.33 (0.95–2.15)	0.071
Not present	Ref	
**Type of toilet used by the household**		
Public toilet	1.35 (0.98–1.87)	0.064
Flush toilet	Ref	
Pit latrine	1.53 (0.81–2.90)	0.193
Bush/river/canal	5.11 (1.79–14.62)	**0.002**
Don’t know	UD(N/A)	1.000

^a^*OR* Odds Ratio. ^*b*^*CI* Confidence interval, *UD* Undefined, *N/A* Not Applicable

Adjusting for other factors, reduced frequency of family eating street food (1 to 2 times/week) was identified as a being protective against contracting *Salmonella* Typhimurium/Enteriditis (aOR = 0.65; CI:0.43–0.98; *p* = 0.041). Additionally, using water pots (aOR = 2.69; CI: 0.98–7.37; *p* = 0.054) and Jerri cans (aOR = 2.63; CI: 0.96–7.19; *p* = 0.059) as water storage containers in the home potentially predicted infection with *Salmonella* Typhimurium/Enteriditis. Adjusting for other factors, male gender (aOR = 1.53; CI:1.17–2.01; *p* = 0.002), age 5–6 years (aOR = 2.49; CI: 1.70–3.65; *p* < 0.001), age 7–8 years (aOR = 2.08; CI: 1.31–3.28; *p* = 0.002) and defecating in the bush/river/canal (aOR = 5.11; 95% CI:1.79–14.62; *p* = 0.002) were predictive of contracting *S.* Typhi. Additionally, adjusting for other factors, age 3–4 years (aOR = 1.43; CI:0.99–2.07; *p* = 0.059), age 9 years and above (aOR = 1.43; CI: 0.95–2.15; *p* = 0.083), keeping chicken (aOR = 1.33; CI: 0.95–2.15; *p* = 0.071) and using public toilets (aOR = 1.35; CI: 0.98–1.87; *p* = 0.064) potentially predicted infection with *S.* Typhi.

## Discussion

We conducted a hospital-based cross-sectional study among children aged < 16 years in one of the major informal settlements of Nairobi, Kenya, in order to determine the prevalence and risk factors associated with salmonellae infection. The prevalence of the major NTS serotypes (*Salmonella* Typhimurium *and S.* Enteriditis) in the population was 1.3% (95% CI: 1.1–1.5%), while that of *S*. Typhi was 1.4% (95% CI: 1.2–1.6%). The male gender, age and defecating in the bush/river/canal were predictive of contracting *S*. Typhi, while keeping chicken and using public toilet was associated with NTS diarrhoeal illness. In addition, socioeconomic status of a family was a major risk factor for life-threatening iNTS disease. It is important to note that Nairobi’s informal settlements where these studies were performed are characterized by dense population, poor sanitation, and unreliable water supply. These are ingredients that create an environment conducive for rapid spread of enteric infections and other sanitation-related pathogens through contaminated food and water [28].

Previous studies from countries such as Qatar [35] and Turkey [36] indicated no statistically significant difference in typhoid prevalence between boys and girls. However, other investigations from Bagladesh [37] found similar findings to ours, with statistically significant higher typhoid infection in boys compared with girls. This perhaps reflects greater exposure of males to contaminated food and water outside the home probably due to their play habits within the slum area where the level of sanitation is low. In our study, higher proportions of infection with *S*. Typhi was observed among children aged 5–6 years and 7–8 years whereas lower proportions were observed among children aged 0–2 years. This could be attributed to the fact that school-going children spend significantly more time playing outside in and around open wastewater trenches. Indirect environmental or fly-based transmission may also have contributed to enhanced disease spread [38, 39]. This is consistent with the finding that infections were higher among children from households that use pit latrine and those who use bush/river/canal as a toilet compared to households using flush toilet. The pit latrines are shared among many households hence increasing the risk of infection through contaminated surfaces in the facility.

The occurrence of iNTS disease was not significantly associated with rearing any domestic animal. Previously it was hypothesized that NTS transmission may be person-to-person rather than through zoonotic reservoirs of NTS bacteria [13, 40]. Rearing chicken was associated with high prevalence of *S.* Typhi (2.1%; OR = 1.75 (CI:1.15–2.70); *p* = 0.011) compared to not rearing chicken (1.4%). Similarly, rearing goats was significantly associated with high prevalence of *S*. Typhi (2.5%; OR = 1.49 (CI:1.15–2.00); *p* = 0.011) compared to not rearing goats. While livestock may not play a role in direct transmission of typhoid, as *S*. Typhi is a human-adapted pathogen, it is likely that keeping livestock is a confounder and may be associated with poor unhygienic conditions in the home.

The proportion of children infected with NTS was significantly higher in households that used water pots as water storage containers compared to those using water directly from the tap. In the informal settlements, water shortage is common and therefore the practice of storing water in pots and plastic containers at home for days before consumption is common. The proportion of children infected with *S*. Typhi was significantly higher among household whose members never washed hands after defecation compared to households where they always practiced hand washing. It is important that people, especially caregivers should always be encouraged to wash their hands after use of a toilet and before feeding, as contamination of hands with fecal matter leads to contamination of surfaces and foods [41].

Globally, it is estimated that *Salmonella* spp. are the cause of over 90 million of diarrhea-associated diseases annually, with 85% of those cases being linked to foodborne sources [42]. Multiple investigations have demonstrated that a considerable proportion of *Salmonella* transmission occurs through contamination along the food chain such as from slaughter of livestock, vegetable and foodcrops harvest to household food preparation [43, 44]. We found that over one-third of the families (38.4%) ate street food three or more times/week, and approximately half of the households bought their food ingredients and vegetables from the shops, with 27.8% buying from mobile vendors and 25.3% from the village market. Consequently, the proportion of salmonella disease was significantly higher among children raised in households whose frequency of eating street food was high. This is corroborated by previous studies in Africa and Asia, which showed that crowded living conditions and poor sanitation were significantly associated with outbreaks of typhoid fever [45–48].

## Conclusion

This study observed that typhoidal and NTS are important causes of illness in children in Mukuru informal settlements, one of the largest informal settlements in the outskirts of Nairobi city. The male gender, age and defecating in the bush/river/canal and using public toilet were important risk factors associated with these diarrhoeal illnesses. The improvement of WASH infrastructure and practices, including boiling water, breastfeeding, hand washing practices, and avoiding animal contact in domestic setting could contribute to reducing the risk of transmission of salmonella disease from contaminated environments common in such settings.

## Data Availability

The datasets used and/or analyzed during the current study are available from the corresponding author on reasonable request.

## Abbreviations

SSA: Sub-Saharan Africa
NTS: Non- typhoidal *Salmonella*
iNTS: Invasive Non-typhoidal *Salmonella*
HIV: Human Immunodeficiency Sydrome
MDR: Multdrug resistant
Pyo: Person-years of observation
NTS D: Non-typhoidal *Salmonella* diarrhea
GIS: Geographic Information System
GPS: Global positioning system\GCPs- Ground Control Points
UTM: Universal Transverse Mercator
KEMRI: Kenya Medical Research Institute
CLSI: Clinical and Laboratory Standards Institute
CHW: Community Health Worker
SERU: Scientific Ethics and Review Unit
OR: Odds Ratio
CI: Confidence intervals
